# Motion Management in a Patient With Tracheostomy During Lung Stereotactic Body Radiation Therapy: Breath Hold Is Worth a Try

**DOI:** 10.1016/j.adro.2022.100895

**Published:** 2022-01-15

**Authors:** Lena Kaestner, Yasser Abo-Madyan, Lena Huber, Manon Spaniol, Kerstin Siebenlist, Marie-Kristin Sacks, Michael Ehmann, Florian Stieler, Sven Clausen, Frank Lohr, Jens Fleckenstein, Judit Boda-Heggemann

**Affiliations:** a Departments of Radiation Oncology, University Medical Center Mannheim, University of Heidelberg, Heidelberg, Germany; b Departments of Otorhinolaryngology, University Medical Center Mannheim, University of Heidelberg, Heidelberg, Germany; c Struttura Complessa di Radioterapia, Dipartimento di Oncologia, Azienda Ospedaliero-Universitaria di Modena, Modena, Italy

## Introduction

Stereotactic body radiation therapy (SBRT) is an appropriate noninvasive treatment option with excellent tumor control rates and overall survival for patients with stage IA non-small cell lung cancer or oligometastases who are medically inoperable or have a high surgical risk.^1^, ^2^, ^3^, ^4^, ^5^, ^6^ To ensure safe dose application during SBRT, a time-resolved motion management strategy is required. In case of breathing-induced uncertainties, possible motion management options include breath hold and free-breathing techniques in combination with either gated or continuous beam-delivery with an internal target volume (ITV) or dynamic tumor tracking.^7^^,^^8^ Deep inspiration breath hold (DIBH) during lung-SBRT leads to expanded healthy lung tissue and tumor immobilization, thus reducing planning target volume (PTV) margins and lung dose compared with ITV-based radiation therapy in free-breathing.^9^, ^10^, ^11^ It can be performed voluntarily or computer-controlled using spirometrical or surface-based systems.^12^ However, a reliable breath hold cannot be performed by all patients, especially when the respiratory capacity is reduced due to comorbidities or previous surgery. In this article, we present the case of a patient treated with lung-SBRT in DIBH for the first time despite previous tracheostomy and reduced respiratory capacity.

## Case Report

A 60-year-old male patient with simultaneous pulmonary adenocarcinoma (cT1b cN0 M0 [American Joint Committee on Cancer IA2] non-small cell lung cancer) and oropharyngeal cancer (cT3 cN2b M0) was presented for initiation of lung-SBRT. Accompanying illnesses included arterial hypertension, bronchial asthma, and advanced chronic obstructive pulmonary disease.

The interdisciplinary oncological conference recommended to treat the head and neck cancer first, followed by resection of both pulmonary lesions as a 2-step process. In accordance with that, the oropharyngeal tumor was treated with surgical resection (pT3 pN0 [0/44] V0 L0 Pn1 G2 R0, marginal carcinoma in situ R1), tracheostomy, and adjuvant chemoradiotherapy (70 Gy and 2 cycles of cisplatin 100 mg/m² q3w). A renewed staging showed no new thoracoabdominal metastases or signs of pulmonary progression. Body plethysmography resulted in an forced expiratory volume in one second of 34% with limited validity due to the patient's tracheostomy. In consideration of the already limited pulmonary function, both lungs being affected, and the ongoing COVID-19 pandemic, the patient was introduced for pulmonary SBRT.

**Fig. 1:**
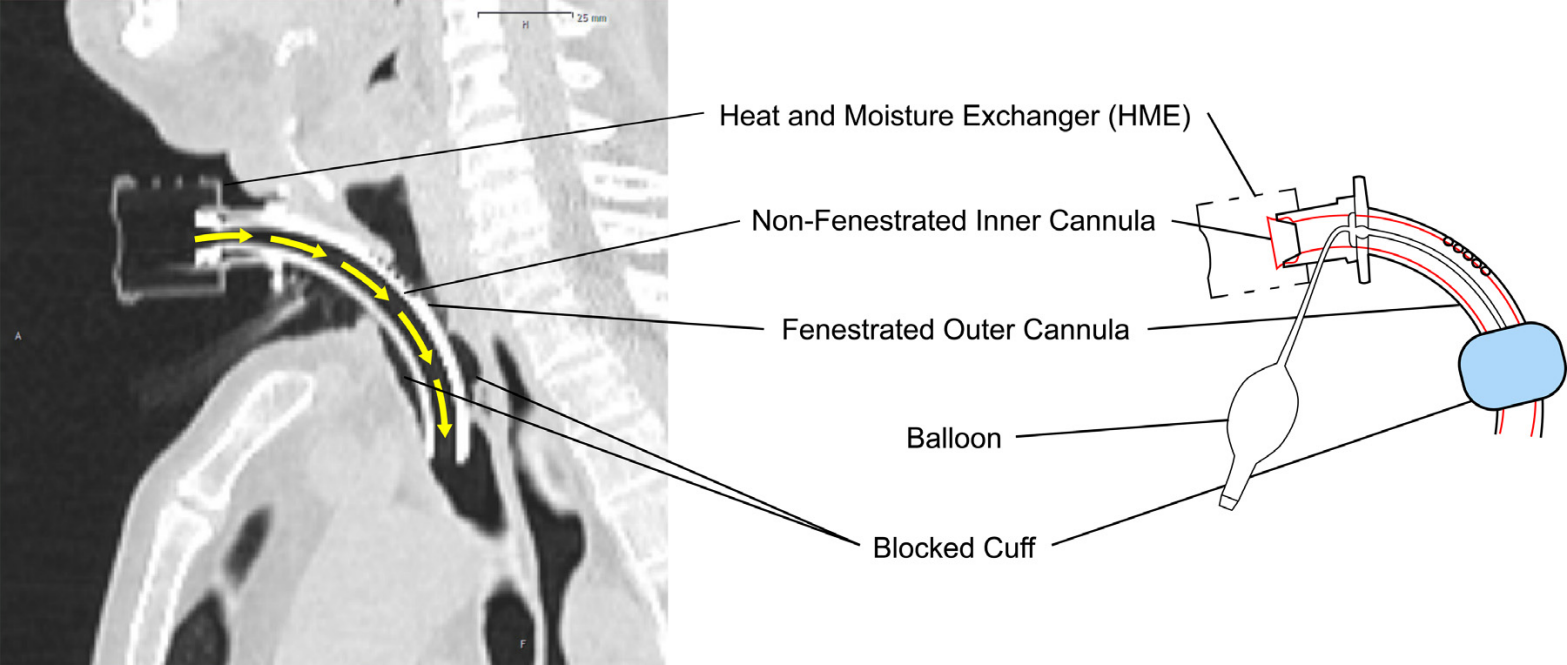
Sagittal computed tomography scan and schematic image of the patient's tracheostoma with a nonfenestrated inner cannula. The cuff was always blocked during radiation therapy and therefore the airflow was directed via the tracheostomy tube (yellow arrows).

At radio-oncological consultation, the patient presented with a body mass index of 21.8 kg/m² and a Karnofsky performance status of 70%. Due to the patient's tracheostomy and reduced forced expiratory volume in one second, a 4-dimensional (4D) planning computed tomography (CT) (Brilliance Big Bore; Philips, Eindhoven, Netherlands) was acquired to account for breathing-induced tumor motion. Due to localization in the lower lobes, the resulting ITVs were relatively large compared with the actual size of each lesion (right/left ITV 11.1/16.5 cm³ vs gross target volume 2.2/3.5 cm³; Table 1). After ensuring that the patient was comfortable with the technique of repeat DIBH despite tracheostomy and that the size of the resulting PTVs and with that the low-dose wash in the volumetric-modulated arc therapy arcs on both lungs could be reduced, we opted for replanning in DIBH. Doses to organs at risk (OARs) were lower in the 4D-CT setup, so that, fulfilling the principle of As Low As Reasonably Achievable, we decided to choose the DIBH plan. To be able to perform DIBH, the patient used his nonfenestrated inner cannula (Ultrasoft-Suction-Voice; Andreas Fahl Medizintechnik-Vertrieb GmbH, Germany) in combination with the already inflated tracheostoma cuff and practiced holding his breath for 20 seconds (Fig 1). Blocking of the tracheostoma cuff was not changed during radiation therapy. Afterward, 10 MV flattening filter free volumetric-modulated arc therapy plans were created with Monaco 5.51 (Elekta AB, Stockholm, Sweden) on both 4D-planning CT and DIBH-planning CT. Dose prescription for the right lesion was 12 × 5 Gy daily (owing to localization in the proximity of thoracic wall; DIBH/4D-CT: D0.5 cm³ [5 Fx <39 Gy] 54.7/60.2 Gy, D30 cm³ [5 Fx <32 Gy] 21.7/31.6 Gy^13^) and for the left lesion 5 × 12 Gy every second day.^14^ Planning constraints were mainly derived from the UK SABR Consortium Report.^13^^,^^15^, ^16^, ^17^ SBRT was performed with the DIBH plans as all dosimetric parameters were more favorable compared with the 4D-CT plans. Summed (left and right lesion) DIBH and 4D-CT plans were additionally evaluated offline (Velocity; Varian, Palo Alto, CA).

**Table 1:** Dose parameters of the summed DIBH versus 4D-CT plans in comparison with OAR constraints for 5-fraction SBRT

		SumPlan DIBH		SumPlan 4D-CT		
Gross tumor volume/internal tumor volume						
Right lower lobe	Volume (cm³)	2.2		11.1		
	D_mean_ (Gy)	61.6		61.3		
Left lower lobe	Volume (cm³)	3.5		16.5		
	D_mean_ (Gy)	83.9		80.9		
PTV						
Right lower lobe	Volume (cm³)	18.1		33.2		
	D2% (Gy)	63.9		63.8		
	D98% (Gy)	57.2		56.7		
Left lower lobe	Volume (cm³)	23.2		43.8		
	D2% (Gy)	87.9		88.9		
	D98% (Gy)	61.1		60.6		
OARs						Constraints (5 fractions)
Heart/pericardium	D_mean_ (Gy)	8.5		10.8		-
	D0.5cm³ (Gy)	28.6		34.2+		29 Gy*
	D15cm³ (Gy)	20.8		26.1		32 Gy*
Total lung	Volume (cm³)	6456.6		5426.7		
	D_mean_ (Gy)	6.5		9.3+		6.5 Gy*
	V13.5 (cm³, %)	1127.8	17.5	1322.7	24.4	37%†
	V20 (cm³, %)	650	10.1+	865.42	15.9 +	10%‡
	CV1500 cm³ (Gy)	9.7		11.6		12.5 Gy*
Chest wall	Volume adjacent to PTV (cm^3^)	110.8		111.6		
	D0.1 cm³ (Gy)	57.3+		61.2+		57 Gy*
	D0.5 cm³ (Gy)	54.7+		60.2+		39 Gy‡
	D30 cm³ (Gy)	21.7		31.6		32 Gy‡
	V37 (cm³, %)	14.6	13.2	24.4	21.8	30 cm³§
Esophagus	D0.5 cm³ (Gy)	12.7		11.7		34 Gy‡
Spinal cord	D0.1 cm³ (Gy)	15.1		14		30 Gy‡
	D1.2 cm³ (Gy)	12.8		11.3		14.5 Gy*

*Abbreviations:* 4D = 4-dimensional; CT = computed tomography; CV = critical volume; DIBH = deep inspiration breath hold; OAR = organs at risk; PTV = planning target volume; SBRT = stereotactic body radiation therapy.

⁎ Gerhard et al.

† Palma et al (SABR-Comet-10).

‡ UK SABR Consortium.

§ Olson et al (Population based phase II trial of stereotactic ablative radiotherapy [SABR] for up to 5 Oligometastases).

**Fig. 2:**
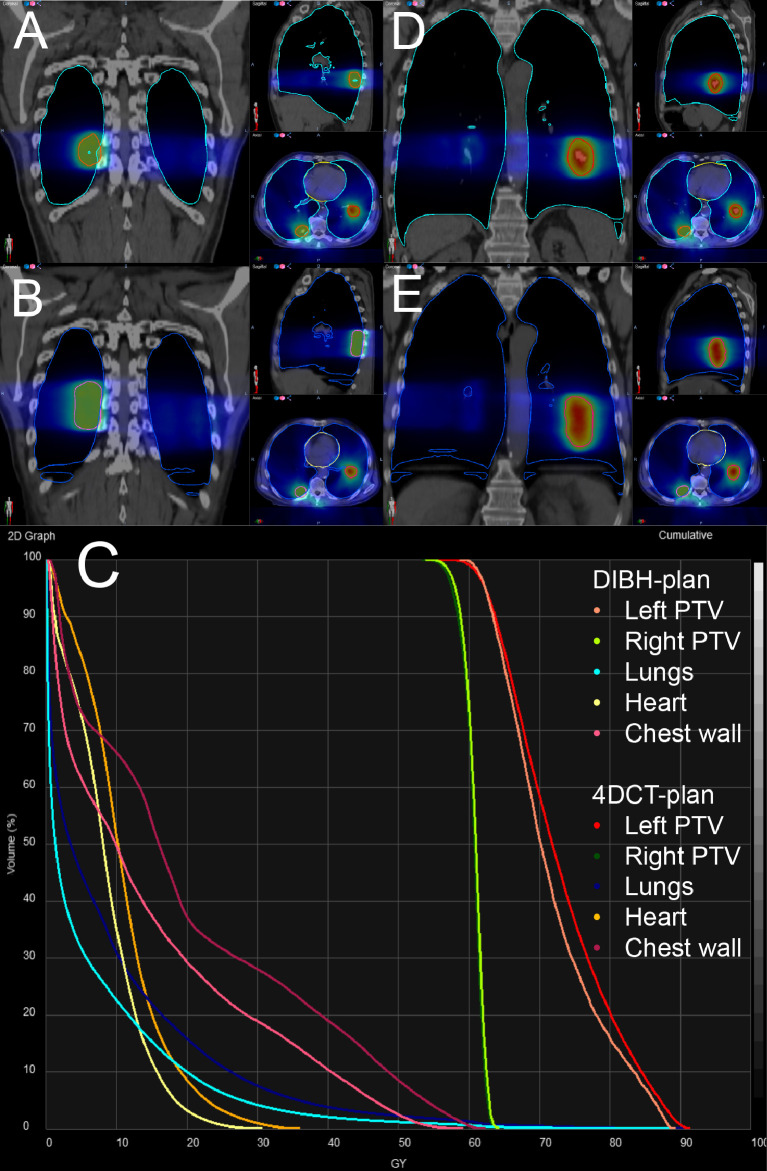
Dose distribution of the summed deep inspiration breath hold (DIBH) plans (A, D) versus 4-dimensional computed tomography (4D-CT) plans (B, E) and dose-volume histogram (C) of DIBH and 4D-CT structures. The curves of the right planning target volumes (PTVs) in DIBH and the 4D-CT plan are almost congruent.

For both DIBH and 4D-CT plans the following dosimetric parameters were analyzed: PTV volumes of the lesion in the right and left lower lobe were 18.1 versus 33.2 cm³ and 23.3 versus 43.8 cm³ for the DIBH and 4D-CT plans, respectively, with similar dose distribution (Table 1, Fig 2). Mean lung dose was smaller in the DIBH plans (6.5 Gy) than in the 4D-CT plans (9.3 Gy) with V13.5 and V20 of 17.5% and 10.1% in the DIBH and 24.4% and 15.9% in the 4D-CT plans, respectively. Dose to the heart was comparable; however, it was also more advantageous in the DIBH plan (D_mean_ 8.5/10.8 Gy and D0.5 cm³ 28.6/34.2 Gy in DIBH/4D-CT plans).

**Fig. 3:**
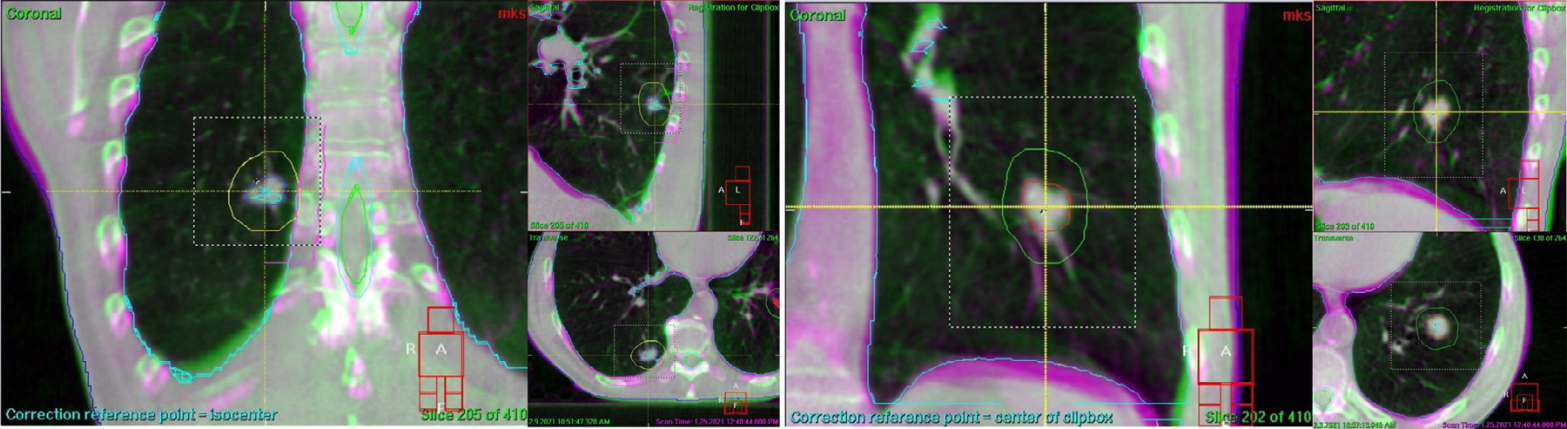
Deep inspiration breath hold–only cone beam computed tomography (CBCT) scans of both targets (pink = planning computed tomography; green = CBCT). CBCT with repeated deep inspiration breath hold has almost a diagnostic quality in tissues with high contrast and makes daily matching more precise than 4-dimensional computed tomography.

**Fig. 4:**
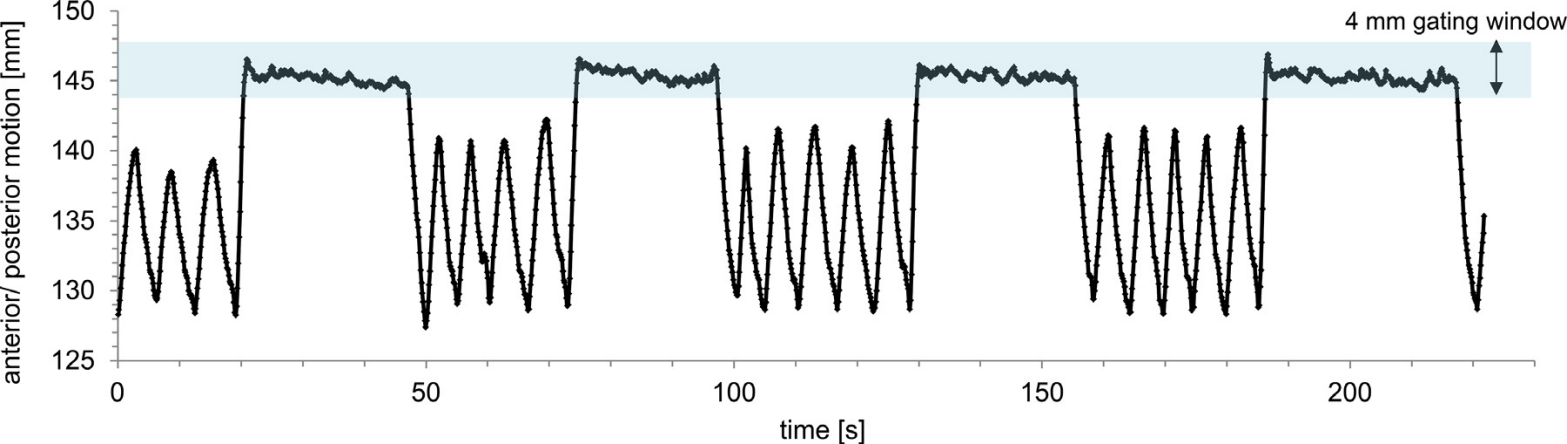
Exemplary breathing motion (anterior/posterior) during deep inspiration breath hold–gated stereotactic body radiation therapy in relation to the planning isocenter detected via a surface scanning system (Catalyst HD, C-RAD). Beam application was only performed if the motion amplitude was in the previously defined gating window. In spite of his tracheostomy, our patient was able to perform a stable, repeated, surface-guided deep inspiration breath hold.

SBRT was performed on a linear accelerator (Versa HD, Elekta AB) in repeated DIBH. The median table time/fraction with 2 SBRT series was 15 minutes.^18^ In advance of each fraction daily image guidance was performed with repeated DIBH cone beam computed tomography (DIBH-CBCT),^19^ as shown in Figure 3. For breath hold monitoring/DIBH gating, the surface scanning system Catalyst HD (C-RAD, Uppsala, Sweden) was used. For SBRT of the right/left lung, mean deviation of the chest wall from the isocenter over all fractions was 198.9 (range, 189.4-202.9 mm)/147.6 mm (range, 144.1-151.3 mm) in anterior/posterior direction. Mean baseline shifts from planning CT to daily surface scanning were 4.1 mm (range, 0.1-6.5 mm) and 4.5 mm (range, 2.1-6.7 mm) for the right and left lung, respectively. Looking at each DIBH individually, maximum range per DIBH was between 1.3 mm and 10.4 mm/between 1.3 mm and 3.4 mm with a standard deviation >1 mm in 4% (range, 0.3-1.3 mm)/0% (range, 0.3-0.6 mm) of all available DIBHs for right and left lung-SBRT, respectively. Only 3 of 69 DIBHs showed few outliers larger than the 8-mm gating window. Figure 4 displays the reproducible motion of the surface with repeated DIBH in this patient despite tracheostomy.

DIBH-based SBRT could be completed as planned and no acute side effects occurred. Both lesions are locally controlled (to the time point of writing the article; Fig E1).

## Discussion

Repeated DIBH is a feasible alternative motion management method for lung-SBRT in tracheostomy patients compared with continuous beam delivery in free breathing. To our knowledge, the case of breath hold gated SBRT in a patient with a protective tracheostomy has never been reported.

The use of DIBH instead of 4D-CT led to reduced target volumes and reduced dose to OARs (mean lung dose 6.5 vs 9.3 Gy, mean heart dose 8.5 vs 10.8 Gy) with comparable PTV coverage. The amplitude of tumor motion depends on localization (greater amplitude in the lower lobe) and whether the tumor is attached to rigid structures.^20^ Therefore, the effect of reduced dose to OAR with DIBH may be either smaller or even larger for different target localizations. DIBH-gated SBRT should especially be considered in tumors with estimated great breathing-induced motion amplitude.

Treatment outcomes for patients with oligometastatic, oligoprogressive, or polymetastatic disease have improved in the last years due to advanced local therapy options and modern systemic targeted/immunotherapies. With that, however, SBRT is more often being combined with drugs, which may result in excess or unexpected toxicity such as fulminant pneumonitis or myocarditis under immunotherapy.^21^^,^^22^ In the last few years, local ablative SBRT has not only been indicated in oligometastatic or in oligoprogressive situations but also for up to 10 or even more metastases.^23^^,^^24^ Additionally, with improving overall survival repeated SBRT series occur more often.^25^ These factors lead to difficulties in obtaining OAR dose constraints. Technical improvements help achieve optimal dose in the target volumes with minimal dose to OARs, enabling sophisticated and ambitious RT plans. Therefore, not only the violation of constraints should be an indication for advanced techniques: In all SBRT cases the most favorable technique should be chosen to minimize dose to OARs.

A spirometrical breath hold system could not be applied,^26^ as it is only designed for oral and nasal air flow. Alternatively, breath hold can be performed and monitored with optical surface scanning systems, pressure belt, or other nonspirometrical alternatives in tracheostomy patients.^27^^,^^28^ The detected amplitude of residual motion during surface-guided DIBH had a maximum range between 1.3 and 10.4 mm per DIBH (standard deviation, 0.3-1.3 mm). This is in line with already published data on residual motion during breath hold in patients receiving lung-SBRT: Koshani et al^29^ measured short- and long-term reproducibility of lung tumor positions during Active Breathing Coordinator-controlled breath hold with a standard deviation of 1.5 and 3.8 mm in anterior-posterior direction. In another study, the intrafractional interquartile range during DIBH-gated surface-guided lung-SBRT was 2.5 mm.^30^

DIBH for this patient was enabled by using a nonfenestrated cannula in combination with the blocked protective tracheostoma. Consequently, the breathing air mainly passed the tracheostoma with no opportunity to close the vocal fold to build up pressure for breath hold. In general, breath holding in patients with tracheostomy can either be performed by sealing the tracheostoma (finger or cap) or imitated by continuously inhaling. In patients after laryngectomy DIBH should not be tried because such a patient is only able to breathe through the tracheostoma and for performing breath hold it would have to be sealed. A very compliant patient like our patient might be able to inhale continuously to reduce motion.

In conclusion, especially for target lesions with a large motion amplitude, DIBH should be considered as an alternative to free breathing/ITV-based target definition when planning SBRT for moving targets (lung, liver, kidney, adrenal glands) in compliant patients with a protective tracheostomy.

## Appendix Supplementary materials

Image, application 1
