# Supplementary figures and images for: Motion Management in a Patient With Tracheostomy During Lung Stereotactic Body Radiation Therapy: Breath Hold Is Worth a Try
Source: Adv Radiat Oncol. 2022 Jan 15;7(3):100895. doi: 10.1016/j.adro.2022.100895 (PMC8850202; doi:10.1016/j.adro.2022.100895)

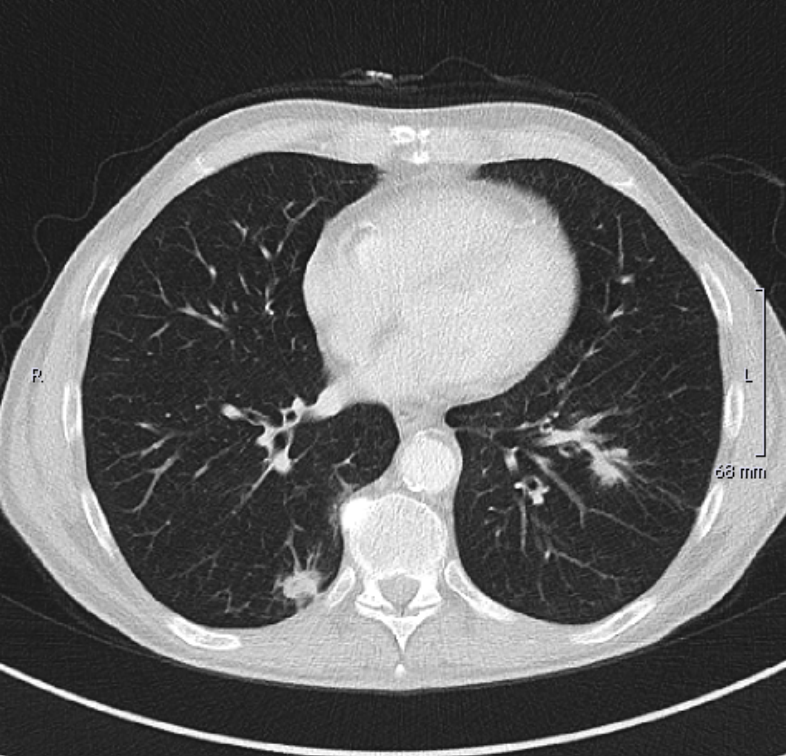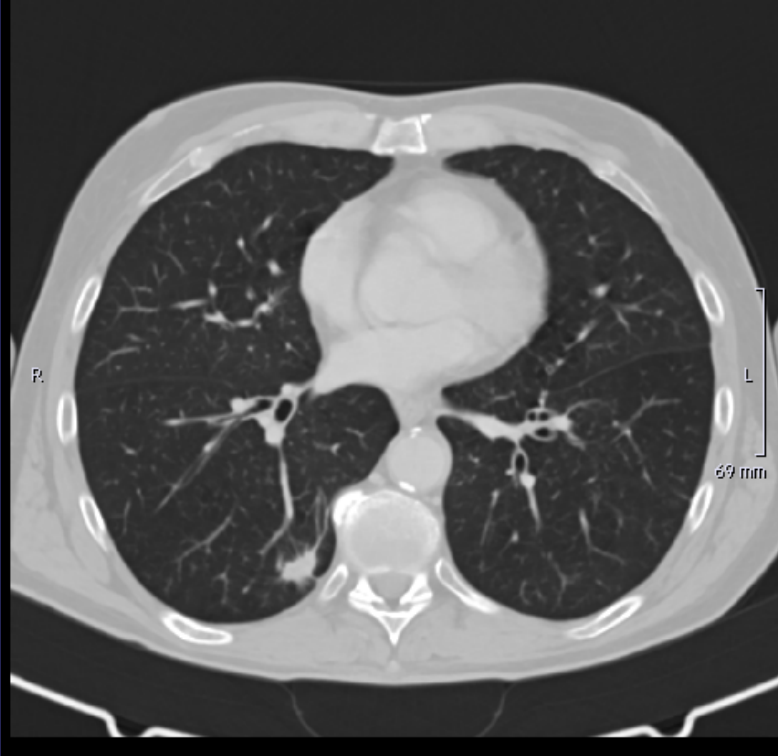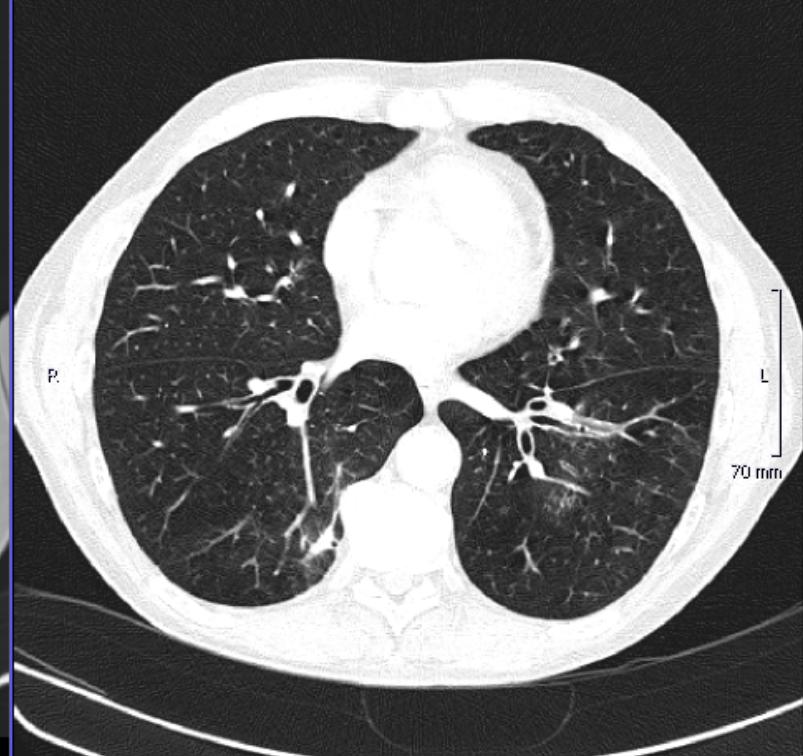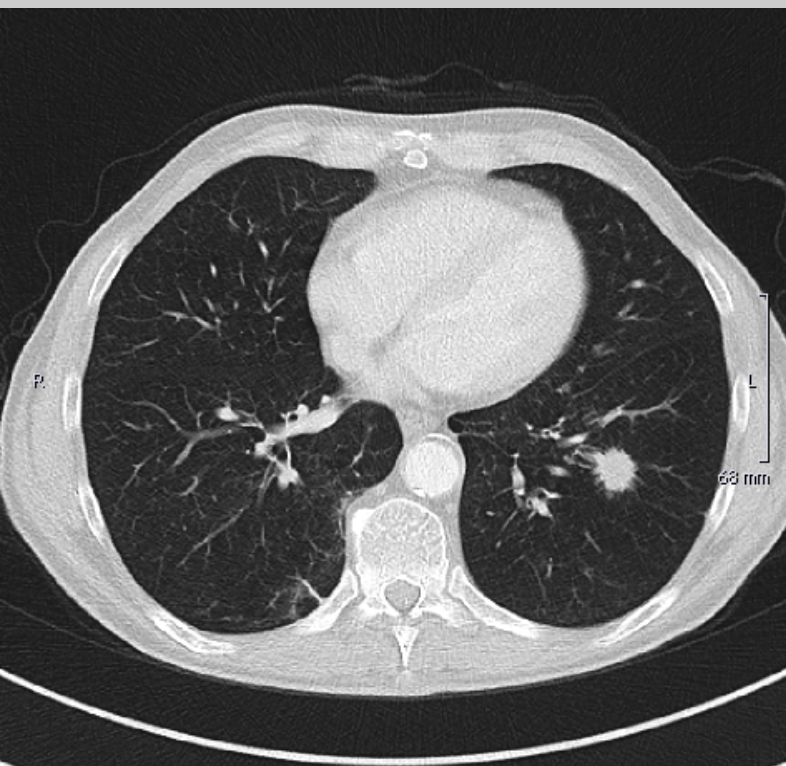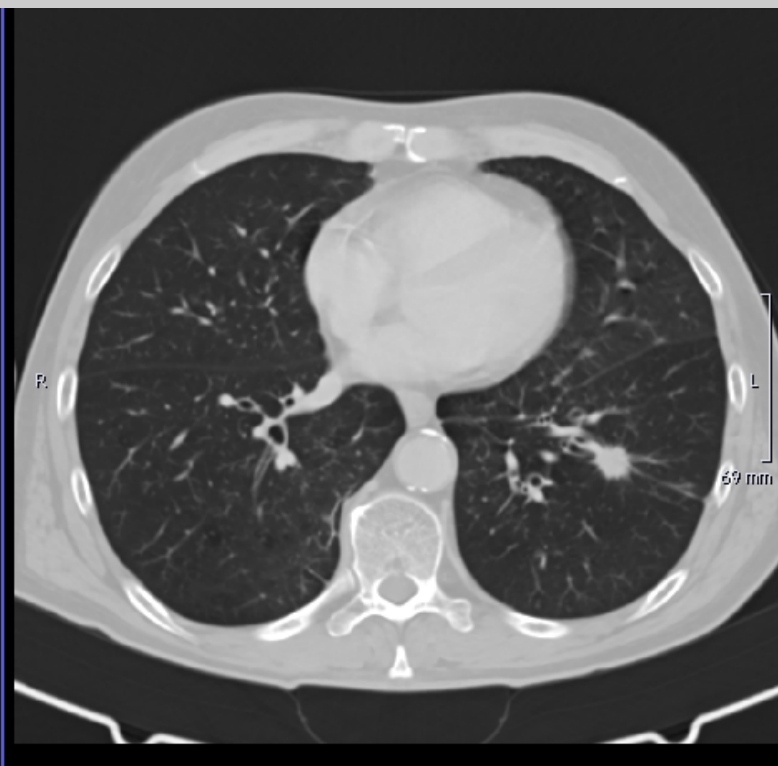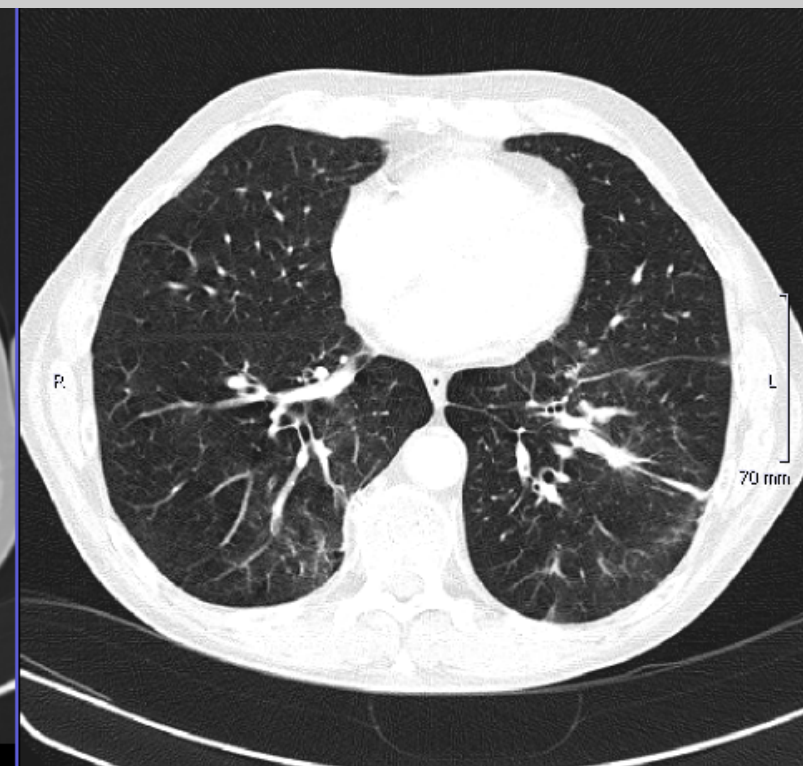

pre-SBRT

6w post-SBRT

5m post-SBRT

Supplement: Supplementary file 1 [file mmc1.pdf]
